# Creature features: The lively narratives of bacteriophages in Soviet biology and medicine

**DOI:** 10.1098/rsnr.2019.0035

**Published:** 2020-01-15

**Authors:** Dmitriy Myelnikov

**Affiliations:** Centre for the History of Science, Technology and Medicine, University of Manchester, Oxford Road, Manchester M13 9PL, UK

**Keywords:** bacteriophage, Soviet science, Lysenkoism, narrative, animacy

## Abstract

The term ‘bacteriophage’ (devourer of bacteria) was coined by Félix d'Herelle in 1917 to describe both the phenomenon of spontaneous destruction of bacterial cultures and an agent responsible. Debates about the nature of bacteriophages raged in the 1920s and 1930s, and there were extensive attempts to use the phenomenon to fight infections. Whereas it eventually became a crucial tool for molecular biology, therapeutic uses of ‘phage’ declined sharply in the West after World War II, but persisted in the Soviet Union, particularly Georgia. Increasingly isolated from Western medical research, Soviet scientists developed their own metaphors of ‘phage’, its nature and action, and communicated them to their peers, medical professionals, and potential patients. In this article, I explore four kinds of narrative that shaped Soviet phage research: the mystique of bacteriophages in the 1920s and 1930s; animated accounts and military metaphors in the 1940s; Lysenkoist notions on bacteriophages as a phase in bacterial development; and the retrospective allocation of credit for the discovery of the bacteriophage during the Cold War. Whereas viruses have been largely seen as barely living, phage narratives consistently featured heroic liveliness or ‘animacy’, which framed the growing consensus on its viral nature. Post-war narratives, shaped by the Lysenkoist movement and the campaigns against adulation of the West, had political power—although many microbiologists remained sceptical, they had to frame their critique within the correct language if they wanted to be published. The dramatic story of bacteriophage research in the Soviet Union is a reminder of the extent to which scientific narratives can be shaped by politics, but it also highlights the diversity of strategies and alternative interpretations possible within those constraints.

Through the course of the twentieth century, bacteriophages have been crucial scientific objects for microbiology, virology, electron microscopy, and molecular biology. Recently, their therapeutic uses against bacterial infections have been receiving much attention and hope, as concerns over antimicrobial resistance intensify. But phage therapy is not new—it is almost as old as d'Herelle's coinage of the term bacteriophage in 1917, and was one among the diverse methods of treating and preventing infection between the world wars. While largely abandoned in the West after World War II, phage therapy persisted in the USSR, particularly Georgia, where a dedicated institute devoted to bacteriophage research and therapy was founded in the 1930s, and exists to this day as the Eliava Institute of Bacteriophage, Microbiology, and Virology.^1^

This article explores the narratives of life, death, and viruses in Soviet bacteriophage research and the histories of the field that Soviet scientists told. While following debates in Europe and the USA, Soviet microbiologists developed unique perspectives on bacteriophages, which were strongly shaped and constrained by political pressures. In the beginning of the Cold War, the campaign against ‘cosmopolitanism’ and the rise of Lysenkoism caused striking departures in biological thinking, but Soviet discussions also had a specific flavour in earlier periods. The mysterious nature of a phage, a ‘substance with creature features’,^2^ and its liminal position between life and non-life made it a fascinating subject and acted as justification for continuous research. Its therapeutic and diagnostic applications, such as phage typing (see Kirchhelle, this issue)^3^, appealed as an example of Marxist science, as phage research combined important theoretical questions with practical applications for the benefit of the Soviet state. Whereas institutional factors were crucial in the survival of phage therapy alongside phage research, the way in which phages were discussed and framed also played an important role.

In analysing the narratives of bacteriophage, I pay special attention to discussions of life and death, and to the language used to describe phages. I am inspired by work on metaphors in science, especially Luis Campos's study of radium as a lively substance—in the first half of the twentieth century, the element was often interpreted as living, owing to its radioactive decay.^4^ Similarly, even though Soviet scientists disagreed over whether phages were living or dead, they continued to be discussed as active agents. In the 1940s, with phage therapy used extensively in the war effort, the framing of phages acquired a strongly militaristic flavour.

Rather than simply asking whether phage had agency in analytical terms, I broaden my examination of phage discourses by drawing on queer theorist Mel Chen's analysis of ‘animacy’ to examine the discourses surrounding phage. Animacy, a concept Chen borrows from linguistic anthropology, is in the first instance a grammatical term, which refers to the likelihood of a noun to be treated as a sentence subject rather than object. Languages and discourses, Chen argues, establish hierarchies of animacy, which do not always map onto the living–non-living binary, but often reveal bias on racial, gender, or other lines.^5^ As I show, despite the equivocal categorization, numerous accounts that made sense of bacteriophages, whatever stance they took, endowed them with animacy and strengthened the argument for phages as living parasites of bacteria. In particular, two different ways of framing phage animacy in the 1950s—an independent virus or a ‘filterable form’ in bacterial development as some Lysenkoists would have it—had consequence not only within the politics of Soviet microbiology, but also in practical terms.

Framing the discussion in terms of narratives allows a more flexible account of scientific ideas and how actors made them cohere, as well as their communication and transit, and their interaction with political and practical worlds of science. Focusing on narratives serves as a reminder that peculiar conceptualizations of phages in the USSR went beyond metaphors—scientists and physicians made strong ontological claims about what phages were, they gave accounts of their reproduction or lack thereof, they produced and reproduced images to make and challenge visual arguments, and they questioned discovery accounts.^6^ In addition, lively narratives influenced practice, as ideas about the nature of phage enabled different experimental interventions and research programmes. Although a detailed account of the practice of phage research is beyond the scope of this article, I offer some examples of phage production during the World War II, and hint at the difference between the practical worlds of Lysenkoist researchers and microbiologists at the Tbilisi Institute.^7^

This article proceeds chronologically while highlighting four kinds of narratives. First, it examines the mystique of phages in the 1920s and 1930s, and the productive role it played in establishing infrastructures and securing research funds. I then examine animacy in the accounts of bacteriophage therapy during the USSR's Winter War with Finland (1940–1941), and during World War II, which the Soviet Union entered when Nazi armies invaded in June 1941. In the post-war years and the early Cold War, Lysenkoist accounts of reversible transformation between bacteria and viruses created a new framework for biologists to work with, and even those critical of the idea needed to make their arguments comprehensible through these dominant narratives if they were to be published. Finally, I examine the official Soviet narratives of the discovery of bacteriophages during the Cold War period—narratives that remained unstable as credit needed to be allocated to Soviet rather than Western scientists, but not to those who had been executed or imprisoned in Stalin's terror. In particular, the complex dynamics of credit and memory played out in the commemoration of Giorgi Eliava, the founder of the Tbilisi Institute which now carries his name. The dramatic story of bacteriophage research in the Soviet Union is a reminder of the extent to which scientific narratives can be shaped by politics, but it also highlights the diversity of strategies and alternative interpretations possible within those constraints.

## The phage mystique

Félix d'Herelle's reports on bacteriophage—referring to both the phenomenon of destruction of bacterial colonies and a putative responsible agent—were first published in 1917, the year of the Russian revolution, but despite the chaos of the civil war they attracted much interest within a few years.^8^ In the 1920s, the new Soviet state embraced microbiology and invested in creating socialized health and expanding its network of bacteriological surveillance. The Pasteur Institute in Paris, where d'Herelle made his observations, played an important role not only as an inspiration for new Soviet bacteriology, but also because a number of prominent Soviet scientists had spent time there. One example of such a visitor was Nikolai Gamaleia, who had published on spontaneous lysis of anthrax bacilli—a precursor to d'Herelle's observations—in 1898, and pursued new work on the phenomenon in the 1920s.^9^ Giorgi Eliava, a Georgian physician and bacteriologist, had established direct contact with d'Herelle during an extended visit to the Pasteur Institute in 1918–21 and became a key proponent of bacteriophage research.

Ever since d'Herelle's announcement, debates raged over the nature of bacteriophage, and whether or not it was ‘alive’.^10^ Its failing to function outside of living bacterial cells suggested an inanimate nature, but its ability to reproduce indicated quite the opposite. Dilution experiments suggested that phages were corpuscular, but some preferred to view them as disorders of bacterial cells. d'Herelle and his allies believed the agent to be a ‘filterable virus’, a parasite of bacteria that could pass through the finest bacteriological filters. Jules Bordet and many others argued for its enzymatic nature and origin within the bacteria instead.^11^ In the 1920s and 1930s, debates in Russian largely mirrored the literature in French, English and German, but a few scientists had more unusual ideas—for example, Gamaleia believed phages to be minute desiccated bacteria, although he abandoned this hypothesis in the 1930s.^12^

While biologists everywhere spent much energy on writing about what life meant, especially when communicating to broader audiences, questions of life, death and immortality had a particularly strong and distinct appeal to Soviet ‘visionary biology’ in the 1920s. Anabiosis, rejuvenation, mysterious rays, and sustained life of isolated organs featured prominently in scientific discussions, brochures, public lectures, and the booming genre of science fiction.^13^ Debates on bacteriophages, although not as prominent as these other themes, reflected some of that fascination. Alexander Oparin, the key figure in speculations on the origins of life from organic molecules, suggested that phages could be fragments of the primordial cells.^14^ Vladimir Vernadsky, a visionary geographer and author of the ‘biosphere’ concept, speculated that bacteriophage was the smallest unit of life, and thus had the highest velocity of spreading ‘biogeochemical energy’.^15^ Nevertheless, whether phages were living or not, and what definitions of life should be used, remained contested and mysterious. In the first original Russian monograph on the subject aimed at a broad readership of biologists, the microbiologist Sofya Kazarnovskaya concluded with a phrase borrowed from her senior colleague Georgy Nadson: bacteriophage was a ‘substance with creature features’ [*veshchestvo so svoistvami sushchestva*].^16^

Meanwhile, even as these lively debates unfolded, several groups of Soviet microbiologists adopted phage for more pressing practical needs, and following d'Herelle's example, pursued phage therapy against a number of diseases, including cholera and dysentery. After some pioneering work in Soviet Ukraine in the early 1930s, led by Moisei Mel'nyk and Hnat Ruchko in Kharkiv, bacteriophage research and therapy acquired a solid footing when d'Herelle himself visited the USSR twice in the winters between 1933 and 1935. Although he was offered his own institute in Moscow, d'Herelle opted for the milder Georgian climate where he could work with Giorgi Eliava.

Eliava was a major figure in Soviet bacteriophage research, and, though he published relatively little, his work as a science organizer and manager proved essential to the expansion of bacteriophage research in the Soviet Union. By recruiting as honourable a guest as d'Herelle, and offering arguments for new treatments—especially for ‘war infections’ such as dysentery and typhoid fever—Eliava managed to secure funding for the major expansion of his Tbilisi Institute into the All-Union Institute ‘Bacteriophage’ in 1935. But just as construction on the building commenced in 1937, Eliava was arrested and executed in the early wave of Stalin's reign of terror. His wife Amelia was also executed, and his adopted daughter Ganna arrested and eventually sent to a prison camp in Kazakhstan.^17^

Bacteriophage research and microbiology more generally gave ample material for the agents of the People's Commissariat of Internal Affairs (NKVD, which became the KGB) to frame their suspects using extensive paper trails and fabrications that accompanied the arrests. Eliava was accused of Georgian nationalism and espionage, recruiting anti-Soviet allies among fellow microbiologists, and various projects of sabotage—faulty bacteriophage and vaccines, poisoning wells with infectious agents, and preparations for bacteriological warfare. In Soviet Ukraine, both Mel'nyk and Ruchko were executed, with similar accusations of nationalism and sabotage.^18^ But the infrastructure investment and growing military needs outweighed any suspicion of phage therapy as such. In 1939, Zinaida Ermol'eva, a microbiologist at the flagship All-Union Institute for Experimental Medicine (VIEM) in Moscow, made a case for further research into bacteriophages as a pivotal biological problem with clear practical applications. Her suggestions appealed to military authorities, embarking on campaigns to invade Eastern Poland and then Finland, in accordance with the secret protocol of the Molotov–Ribbentrop pact between the USSR and Nazi Germany.

## Military metaphors

It was during the Winter War with Finland (1939–1940) that bacteriophage therapy was tested against wound infections. The trials were conducted by a Tbilisi team headed by the surgeon Alexander Tsulukidze, and Leningrad microbiologists led by Magdalina Pokrovskaia, who had experimented with plague phages in the 1930s.^19^ In their accounts of these trials, which they deemed a qualified success, as well as in medical guidance and patient diaries and memories, bacteriophages are mostly treated as another medicine. Available as liquid solutions and sometimes as powder during the Winter and Great Patriotic Wars, phages acted as substitutes for sulfa drugs and later penicillin, neither widely available in the USSR during World War II. Yet, when it came to the nature and action of phages, another pattern emerges from the discussions in these papers. Most authors declared uncertainty over the nature of phages, but the language they used to discuss the mysterious agent was highly animated.

Military metaphors were nothing new in bacteriology, and by no means unique to the Soviet context. Discussion of germ theory and immunity abound with images of battles, infiltration, counterattacks and victories. Promising antimicrobial drugs such as salvarsan and later penicillin were famously described as ‘magic bullets’.^20^ But whereas medicines such as penicillin or gramicidin were usually analogous to weapons, bacteriophages came with more elaborate and animated metaphors. They were variously described as agents, spies and armies that destroyed bacterial cells from within, even by authors who were uncertain as to their nature.

As a collaborator of Eliava and d'Herelle, Tsulukidze shared their idea about the viral nature of phages. Without any qualms, he declared that studies on the phenomena came to ‘certain conclusions’: that bacteriophage was a ‘living principle’ [*zhivoe nachalo*], a ‘filterable virus’, an obligatory parasite of bacteria with a corpuscular structure. With this picture in mind, it is not surprising that Tsulukidze described bacteriophage in language suggesting clear agency: it ‘dissolved’ or ‘lysed’ bacteria and it ‘reproduced’. Bacteria, in turn, were ‘infected’ by bacteriophage and were either destroyed or ‘became “ill”, non-viable, and lost their virulence’.^21^ Tsulukidze's Leningrad colleagues in the Finland War trials, led by Magdalina Pokrovskaia, were far more equivocal about the nature of phage, citing d'Herelle's virus model but treating it as unproven. Accordingly, in their writing they discussed bacteriophage as a phenomenon and a substance with ‘viability’, specificity and ‘virulence’, but, when presenting d'Herelle's views, relied on curious metaphors. Bacteriophage thus ‘stuck’ to the bacterial surface, then ‘infiltrated’ [*pronikaet*] and started to produce a special enzyme called lysin. As a result, new ‘young’ bacteriophages were freed from the cell as ‘embryos’ [*zarodyshi*].^22^ They could also be ‘gradually trained’ to adapt to different environmental factors, such as higher temperatures.^23^ Overall, Pokrovskaia *et al.* shared the ‘substance with creature features’ perspective, expressing hope that better understanding of bacteriophages could ‘fill the void which still lies between living and dead nature.’^24^

Similar ambiguity over phage animacy showed itself in discussions of the practice of phage research. Whereas in many ways phage solutions were treated as a chemically or biologically derived medicine—in terms of storage, dosage, delivery regimen—other practical interventions implied living agents interacting with the bacteria they destroyed. Involvement of d'Herelle's private laboratory in phage production and his strong views on recommended protocols, based on the assumption of phage as a living bacterial parasite, had an effect. Thus, Pokrovskaia suggested that the ‘virulence’ of a low-efficiency phage could be improved if it were passaged through bacteria—that is, used to destroy a series of different bacterial cultures and filtered each time.^25^ Passaging phages through cultures and patients, and developing practices to sustain phage strains and maintain their virulence, meant approaching the mysterious phenomenon as living, at least in those engagements with it.

Though Pokrovskaia's readership may have been largely limited to physicians and enthusiastic military authorities, her team's ideas and metaphors circulated more widely, for instance in *A treacherous weapon*, a 1942 novella about bacteriological warfare aimed at teenagers. Subtitled ‘a non-fantastic story’, it was written by Sergei Beliaev, a physician and science fiction writer, under the pseudonym E. Kramskoi.^26^ In two parallel stories, German spies plan trials to spread germs in Paris, while two Soviet scientists anticipate the attack as they develop the ultra-powerful optical ‘needle microscope’ that would allow real-time *in vivo* observation. Bacteriophages appear in one of the rather didactic asides in which both heroes and villains discuss pathogens, the nobility of bacteriology, and defences against bacterial warfare.^27^ Beliaev's description of bacteriophages featured bacterial lysins and reproducing ‘embryos’, and was strikingly similar to Pokrovskaia's language—Beliaev must have read her team's book.^28^

Later books and brochures published on phage maintained the mystique over its nature, and emphasized its liminal place between life and death. Literature aiming at a wider audience, including medical practitioners, students and patients, was bolder with its metaphors, but maintained a similar pattern when discussing the phenomenon. The vast majority adopted ‘devourer [*pozhiratel’*] of bacteria’ as a translation of ‘bacteriophage’, despite d'Herelle's reported unhappiness with this reading.^29^ In a 10-page brochure for the Leningrad House of Sanitary Education, another Leningrad-based microbiologist, Moisei Fisher, was careful to emphasize the uncertain nature of bacteriophage, acknowledging that ‘many scientists, including our Soviet ones, consider bacteriophages to be the products of bacteria themselves and not self-sufficient creatures.’^30^ But the brochure was titled *Bacteriophage: the devourer of microbes*, and, when discussing the phenomenon itself, Fisher wrote of a lively agent performing a number of actions. Bacteriophage ‘dissolved’ bacteria, although sometimes microbes get ‘ill’ and lose toxicity. It ‘infected’ bacteria as dangerous as the cholera vibrio and plague bacilli. A well-made, strong bacteriophage could ‘neutralize’ or ‘disarm’ [*obezvrezhivaet*] the microbes and ‘liberate’ the patient's organism from them.^31^ Thus, while maintaining ambiguity about the nature of phage, these narratives imbued it with agency and specificity, supporting the view of the phage as a virus. They also aligned phages with Soviet military prowess.

As World War II progressed, Western debates over the nature of phage were settling down, especially as accounts of lytic/lysogenic cycle were elaborated by Burnet, Delbrück and others.^32^ In the process, the meaning of ‘virus’ also changed—from being defined through direct link to the disease they were causing, and by their effect on bacterial, plant or human cells, viruses were becoming their own particles with structure, morphology and classification.^33^ Electron microscopy made viruses visible for the first time, but its role was by no means straightforward. Proponents of the new tool spent much energy distinguishing observations from artefact, and relied heavily on calibrating new images against existing visual knowledge—severely lacking in the case of viruses.^34^

**Figure 1.**
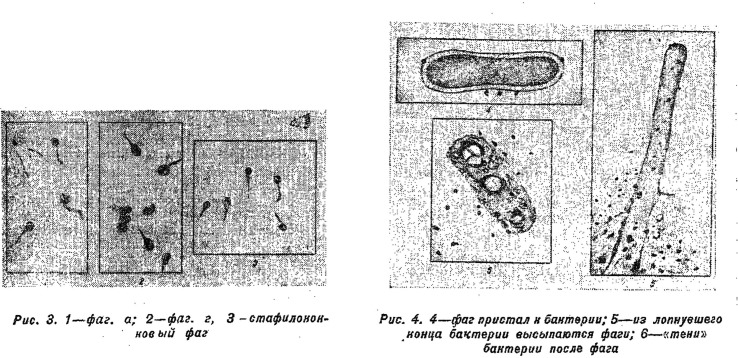
Early drawings of bacteriophages destroying a bacterial cell, based on electron micrographs, to appear in Russian periodicals. Klein, *op. cit.* (note 35), p. 37, caption reads: ‘Drawing 3. 1—phage a; 2—phage g; 3—staphylococcus phage; Drawing 4. 4—phage attached to a bacterium; 5—phage pour out from the burst end of a bacterium; 6—“shadows” of a bacterium after phage’. (Reproduced courtesy of Nauka i Zhizn’.)

The power of visual arguments was strong and Soviet researchers paid attention to developments in Germany and the USA. Images alone, however, were insufficient to settle the debate.^35^ In 1945, drawings based on the electron micrographs of bacteriophages made appearances in the general-audience *Nauka i Zhizn’* [*Science and life*].^36^ Boris Klein, reproduced images of tiny phages attaching to a bacterium, followed by a destroyed cell with more phages emerging, as well as more zoomed-in images of ‘sperm-like’ phage particles (figure 1). While acknowledging the images supported d'Herelle's hypothesis, Klein did not present them as decisive arguments, especially as the ideas about viruses remained ambiguous. He saw the images consistent with his idea that phages were ‘internal antagonists’ of bacterial cells, or enzymes responsible for lysis.^37^ Similarly, Anatoly Kriss, writing earlier that year in the USSR Academy of Sciences flagship journal *Priroda* [*Nature*], viewed the new images as consistent with his ideas about phages being complex enzymes.^38^

After World War II, penicillin and other antibiotics soon eclipsed bacteriophages as antimicrobial interventions, though phage therapy persisted on a smaller scale.^39^ The electron micrograph images directed attention away from medical applications back to the questions of phage structure and nature. Yet the military metaphors that accompanied medical discussions of phage therapy did not disappear after the war. In 1949, an article in *Tekhnika—molodëzhi* [*Technology for the youth*] introduced further advances in electron microscopy and what they revealed about the ‘microworld’:

Bacteriophage, a creature alien to the [human] organism, but its loyal ally, had been invisible, incomprehensible and mysterious, which is why its “military operations” had been, until recently, entirely unknown. But then the electron microscope successfully acted as a photojournalist, and its remarkably valuable photographic report demonstrated the fascinating episodes of the battles between bacteriophage and bacillus.^40^

Mel Chen's work on animacy is based on the premise that ‘matter that is considered insensate, immobile, deathly, or otherwise “wrong” animates cultural life in important ways.’^41^ Ambiguity over the lively status of a substance further adds to the impact on cultural readings of a phenomenon. During the Winter War and World War II, animated discussions about bacteriophages made their living and viral nature more palatable and convincing, aided by military connotations that offered convenient contemporary ways to conceptualize viruses infecting bacteria. While electron micrographs were not straightforwardly received, they also offered support to the parasite model, and the end-of-war engagement with the electron microscopy data happened at the peak of cooperation between Soviet and Western European and US science, and therefore new models of phage biology. Yet as the relationship between the USSR and its formal allies deteriorated in the late 1940s, narratives of phage nature moved on a distinct trajectory.

## Phages and Lysenkoism

After the end of World War II, the Cold War soon unfolded, as former allies divided Europe in the shadow of the atomic bomb. Former affinities were replaced by suspicion and tension in a number of spheres, and, as Nikolai Krementsov has shown, biomedical sciences played a significant role in Cold War diplomacy.^42^ On the one hand, this development was not necessarily detrimental to phage research and especially therapy in the USSR—indeed, the growing isolation protected Soviet phage therapy from outright dismissals in the British and US medical press, even if its therapeutic significance did decline.^43^ On the other hand, new political regimes created new challenges for phage researchers, as they had to navigate the terrains of Soviet self-aggrandisement in assigning scientific credit, and especially the growing dominance of Lysenkoism in the life sciences.

Trofim Lysenko's vision of heredity, which disavowed Mendelian genetics and especially its gene-mapping approaches most associated with T. H. Morgan's school, grew to dominate Soviet biology after World War II. The 1948 session of the Lenin All-Union Academy of Agricultural Sciences (VASKhNIL) was a landmark event that cemented his influence and made genetics a dangerous activity.^44^ The elaborate and high-strung discussions touched on many topics, bacteriophages among them. In a bold defence of genetics and the materiality of the gene, the geneticist and war hero Iosif Rapoport appealed to the new images of bacteriophages made possible through electron microscopy. Phages, whose existence many scientists had ‘denied until recent days,’ could now be observed despite their small size; therefore the gene, ‘an even more mysterious unit’, would also be demonstrated as a material, not merely metaphysical, entity.^45^ In response, loyal Lysenkoist and former NKVD agent Sergei Muromtsev decried,

What did Professor Rapoport mean when he said that we could see bacteriophage with the aid of an electron microscope? As I understand, he thinks this is a decisive proof that phages are living organisms. Not everything we see is living, Professor Rapoport, that's the first thing, and second, the corpuscular nature of phages has long been proven …^46^

As Lysenkoism took firm hold over Soviet biology, microbiology, like other disciplines, experienced a ‘Michurnist’ turn, named after the agronomist-turned-Lysenkoist-hero Ivan Michurin.^47^ Muromtsev's 1950 lecture on the ‘Problems of modern microbiology in the light of Michurinist doctrine’ gives some idea of what this turn entailed. Muromtsev focused on microbial variation, and attacked recent developments in bacterial genetics, especially the celebrated work of Salvador Luria and Max Delbrück on phages and bacterial mutations as examples of natural selection. Instead, he cited prominent Russian microbiologists such as Elli (Il'ia) Metchnikoff, Sergei Vinogradsky and Nikolai Gamaleia, as supporters of the environment's ability to transform bacterial species. Soviet researchers, claimed Muromtsev, developed these insights by grounding their work in dialectical materialism, ‘the great teachings of Marx–Engels–Lenin–Stalin’, and generated numerous practical as well as theoretical advances. In general, Michurnist microbiology denied any special hereditary material, but posited that species transformed by accumulating environmental changes, as quantity transformed into quality when new species formed, in keeping with dialectical materialism.^48^

A Michurinist doctrine developed by Olga Lepeshinskaya had special relevance to phages. Lepeshinskaya, who worked in cell biology and embryology, postulated acellular forms of ‘living matter’ that could, under certain conditions, develop into cells. Her work focused on animal cells and embryos, and the argument had especial relevance to viruses and bacteriophage.^49^ Between 1948 and 1952, Michurinist microbiology developed along new lines suggested by Lepeshinskaya and a maverick veterinary microbiologist, Gevork Bosh'ian, whose 1948 monograph, *On the origins of viruses and microbes,* claimed dramatic new discoveries.^50^ Bosh'ian postulated transformation of viral particles into bacterial cells and vice versa:

d'Herelle's enormous achievement was in recognising the living nature of bacteriophage …. To this day, most microbiologists erroneously insist that bacteriophage is an independent parasite that has nothing in common with the microbe, from which it is formed …. Our research shows that bacteriophage is a phenomenon of decomposition, disintegration of the bacterial cell into particles whose size lies beyond the resolving power of our regular microscopes.^51^

These particles could, in turn, assemble into bacteria, and represented ‘filterable forms’, a phase in microbial development.

Whereas Bosh'ian cited neither Lysenko nor Lepeshinskaya, the latter promoted his work as proving her theory of inanimate matter, and it received favourable reviews in medical and lay periodicals. The second edition of his book, published in 1950, had an astonishing print run of 100 000 copies. Ideas of viruses as ‘filterable forms’ of bacterial development attracted new allies. Thus, in 1952, a group of scientists at the Moscow Institute of Microbiology, Epidemiology and Infectious Diseases argued that bacteriophage was a bacterial precursor, an argument that mirrored Gamaleia's 1920s speculation.^52^ Electron microscopy was not incompatible with this view of bacteriophage—indeed, most proponents recruited the new images as evidence, and called for better equipment and techniques.

Bosh'ian's reading of viruses did not last long, in part owing to the ambition of his subversive claims, but in part because he failed to secure the most important allies. Lysenko was cautious in endorsing Bosh'ian, and critical of his book in private, and his microbiologist ally Muromtsev criticized this novel interpretation of bacteriophage as ‘not in agreement with the facts’.^53^ Anti-Lysenkoist biologists were more damning in their reviews, and even those who tried balancing Michurnist language with traditional microbiology attacked the theory. Thus, Alexander Krivisky, while using the language of ‘filterable forms’ and lauding the ‘brilliant research’ of Lepeshinskaya, argued for the understanding of lysogeny as a dormant phase of phage development, and of bacteriophage as a viral parasite.^54^

Much of the debate revolved around researchers in Moscow. In the relative isolation of Tbilisi, the pressure to pursue Lysenkoist programmes was much weaker. At the Tbilisi Institute of Microbiology, Epidemiology and Bacteriophage (IMEB, as Eliava's brainchild was named in 1937), a new generation of microbiologists focused on studying microbial variation through an ecological lens. Michurnist microbiology was not completely absent from the Institute—for instance, a junior researcher, Nikolai Bystry, reported on the latest developments to the Institute's scientific council. In his account, he urged colleagues to interrogate the concept of species, both of phages and bacteria, by intensifying work on microbial variation—a subject of great interest in the pre-war years, and one closely linked to bacteriophage research.^55^

Tbilisi IMEB may have claimed a Michurinist–Lysenkoist orientation in formal reports, but the influence of Bosh'ian, Lepeshinskaya and Lysenko were minimal. Indeed, an inspector criticized the Institute for not paying sufficient attention to ‘creative Darwinism’.^56^ Headed by Eliava's former assistant, Elena Makashvili, the bacteriophage department continued to frame its work ecologically, developing d'Herelle's view of phage as important in recovery and human immunity.^57^ In 1951, she stated that ‘considering the living nature of bacteriophage, the Institute bases its work on the principle of an unbreakable bond between the living organism and its environment.’^58^ How bacteriophages behaved in human bodies, how they could be strengthened or weakened by their environments, and the role they played in bacterial variations were the key research questions. At the same time, while the framing of research in ecological terms allowed new avenues for research, it also prevented scientists in Tbilisi from engaging with the latest findings on bacterial heredity, such as the work performed by Luria, Delbrück, and the Phage Group in the USA, at least until the late 1950s. Furthermore, access to foreign literature had been problematic, as periodicals and books from overseas had not been supplied to the library since 1939, and could only be obtained via interlibrary loan.^59^

Michurinist microbiologists and researchers at the Tbilisi IMEB proposed two different modes of phage animacy, whereby the mysterious phenomenon was assigned a different place within the lively hierarchies. For Georgian microbiologists, phage was an independent bacterium-destroying agent, resisting and fighting bacteria, and perhaps playing a role in recovery, whereas for experimentalists working with Bosh'ian's ideas, it was a continuous step in bacterial development. The two models of animacy had practical implications for the kinds of experiments their proponents pursued. Researchers invested in the ‘filterable form’ model sought to demonstrate continuity between bacteriophages and bacteria, using serological methods (i.e. antibodies) to show that both bacterial cells and isolated bacteriophage could bind to the same specific probe.^60^ Scientists at the IMEB, by contrast, examined the relationship between phages and their bacterial hosts, used phages to type bacterial strains, examined the effects of dysentery patients’ gut microflora on the efficiency of phage therapy, and even tried to use phages isolated from recovered patients rather than bacterial cultures.

Soviet debates around the nature of bacteriophage in the 1950s highlight the extent to which biological knowledge was shaped by political pressures. Although Bosh'ian and Lepeshinskaya's views were not unanimously accepted, they defined the framings of a major issue. Furthermore, in isolation from much international research, Soviet microbiologists developed a series of unique narratives on the nature of bacteriophage. At the same time, there was a certain amount of flexibility in reading and manoeuvring among these accounts, and tensions in microbiology were less extreme than in genetics proper. Location on the periphery could be an asset, but it also meant that research agendas had to follow certain paths. Yet it was not only the nature of phages that was contested in the 1950s—on the wave of isolationism and the campaign against ‘cosmopolitanism’, scientists’ histories of phage research and allocation of credit were being rewritten, too.

## Credit and memory in the Cold War

As I have shown, debates around bacteriophages were volatile in the 1950s, driven by Lysenkoist readings and resistance to them, as well as attempts to negotiate alternative meanings of the phenomenon through an ecological perspective. But the nature of phage was not the only issue in question—narratives around its discovery and the assignment of credit also acquired new political meanings. The consequences of Stalin's Terror and silence around the victims erased contributions of Eliava, Ruchko and others, and while they were in Khrushchev's Thaw in the mid-1950s, scientists were slow to rewrite the redacted histories. In the early years of the Cold War, before Stalin's death in 1953, issues of credit went beyond those murdered by the state—as the USSR ramped up isolationism and the fight against ‘cosmopolitanism’, ‘adulation’ [*nizkopoklonnichestvo*] of foreign science was a new sin, and credit for discoveries had to be reallocated retrospectively.

When the IMEB was being reviewed by the Moscow authorities in 1949, its lack of committed engagement with the ‘creative Darwinism’ of Michurin–Lysenko was not its only fault. The inspector, comrade Sokolov, demanded other improvements to the ‘ideological work’:

some of our scientists ignore the discoveries of our compatriots and attribute these achievements to foreign scientists … everyone now knows that our scientist Gamaleia discovered the bacteriophage phenomenon, while this is being attributed to d'Herelle. We must unleash the fight for the priority of Soviet Science, against cosmopolitanism and adulation of foreign things [*pered inostranshchinoi*], while simultaneously aiming for in-depth study of Marxist–Leninist biology and Soviet creative Darwinism.^61^

Gamaleia, who had died shortly before the inspection, was thus promoted from one of the precursors who had ‘anticipated’ bacteriophage with his 1898 observation on the spontaneous lysis of anthrax bacilli, to the discoverer of bacteriophage. Gamaleia represented both a direct link with the foundation of germ theory—he had worked with Pasteur and Metchnikoff in Paris in the 1880s—and also a model Russian and Soviet scientist. But his case was not unique even in microbiology—thus, as the USSR failed to acquire US penicillin after World War II, as relationships between the countries deteriorated, much was invested in developing a Soviet analogue, *krustozin*, isolated by Zinaida Ermol'eva's group during the war.^62^ With the (ultimately unsuccessful) transition to domestic penicillin, the allocation of credit also changed. In 1948, *Pravda* declared that ‘Penicillin is a Russian discovery,’ citing Ermol'eva as well as Manassein and Polotebnov, nineteenth-century Russian mould researchers who had observed its antibacterial effects.^63^

After Stalin's death in 1953, Khrushchev's Thaw enabled new connections to international exchanges, and while the new Secretary General supported Lysenko, the hold of Bosh'ian and Lepeshinskaya on microbiology weakened. In the same year, Tbilisi IMEB was renamed as the Tbilisi Institute of Vaccines and Sera (TIVS), removing bacteriophage from its name. In 1952, the USSR Ministry of Health had removed the issue of bacteriophage from the list of the key research problems, and the pursuit of new antibiotics eclipsed interest in phage therapy in most Soviet institutions. In 1955, however, by appealing to the local and All-Union authorities and responding to local dysentery outbreaks, leaders of the Tbilisi Institute succeeded in re-establishing the importance of phage therapy, as well as the tools of phage typing, and organized a major conference on the problem of bacteriophage. In the aftermath of the meeting, attended by microbiologists from across the USSR, TIVS was assigned the methodological centre for bacteriophage, which meant it was to coordinate research on the topic across the microbiology institute network, develop a standard phage collection, and supply bacteriophages for research, treatment and typing needs.

Opening the 1955 conference, Vladimir Antadze drew parallels between the Institute's tenacity and improving fortunes to a ‘lysogenic line’ of phage, lying dormant inside a bacterium until the conditions were right. Lauding the Institute's ‘pioneering’ role in the history of Soviet bacteriophage research, he liberally quoted d'Herelle, with whom he had worked in the 1930s. Recent experiments with radioisotopes and electron microscopy, Antadze argued, clearly vindicated d'Herelle's hypothesis that the bacteriophage was a viral parasite of bacteria.^64^ Yet while d'Herelle's name returned as a subject of pride, Eliava remained absent. In 1957, Antadze authored an article on the history of microbiology in Georgia for the flagship *Journal of Microbiology, Epidemiology and Immunobiology*, in which he claimed: ‘Georgian microbiologists are the pioneers of developing the problem of bacteriophage in the Soviet Union. This has been aided by scientific connection between F. d'Herelle and *the collective of the Tbilisi Institute of Epidemiology and Microbiology*, which began in 1921 and has continued throughout’ [emphasis added]. Euphemistically, Eliava's role was concealed.

The omission is not surprising—Eliava was only formally rehabilitated in August 1957, two months after Antadze had submitted his piece. Yet the management of the Tbilisi Institute avoided official mentions of Eliava in the following years. While Khruschev's rehabilitation of the dead did not translate into automatic return into textbooks and official histories, other repressed scientists had been acknowledged by their institutions. The microbial geneticist Georgy Nadson, for instance, was posthumously reinstated as a member of the Academy of Science of the USSR in a public vote at a general meeting shortly after his formal rehabilitation in 1956.^65^ But rehabilitation and commemoration were especially difficult in Georgia, where Stalin's legacy became highly contested after the landmark 20th congress of the Communist Party in 1956.^66^ Memory of the dead and imprisoned clashed with the dictator's Georgian origins, and there were major protests against Khrushchev's de-Stalinization programme; in March 1956, these protests culminated in riots in Tbilisi.^67^ As David Shrayer-Petrov recalls, during his visit to the Tbilisi Institute in 1959, ‘people were still very reserved, afraid to touch upon any political issues, and there was a presentiment of the possibility of a return to that earlier, reactionary period.’^68^

The first post-war mention of Eliava in print known to me was in *Viruses against microbes*, a 1962 general-interest book on bacteriophages by Krivisky, whom we have seen navigating between Michurinist microbiology and the Western consensus on viruses in the 1950s*.* Free of these pressures after Lysenko's fall from grace, *Viruses against microbes* communicated the recent discoveries of Western molecular biology on the structure of viruses and phages alongside Soviet contributions, recounted d'Herelle and Eliava's collaboration, and even featured a photograph of Eliava, noting that it was being ‘published for the first time’.^69^ Krivisky, however, was based in Moscow, and it was only in 1974 that Eliava received full credit as the founding figure of his institute.^70^ Celebrating 50 years since the foundation of what was initially the All-Union Institute ‘Bacteriophage’, the director and Eliava's former employee, Irakly Georgadze, firmly re-established Eliava's place in the official narrative. Georgadze had pursued archival research trying to reconstruct Eliava's biography and role in the establishment of bacteriophage research in Georgia, and while he had to resign to a euphemism when referring to Eliava's ‘untimely death’, the extent of biographical information was great. Elena Makashvili and Eliava's daughter Ganna, who had survived prison camps after her parents’ arrest, were involved in the celebrations and possibly the writing of the piece.

It is difficult to know whether Eliava's memory remained silenced through the late 1950s and early 1960s owing to institutional management's choice, local censorship, or the general fear that accompanied discussions of Stalinism. Eliava's former associates, indulging Makashvili and Georgadze, kept fond memories of him. The clearest indication of the continued quiet local memory can be found among the depressing paper trail of the 1937 case against Eliava, in which one document stands out. During the rehabilitation proceedings in 1956–7, Nina Egiazarova, another of Eliava's former technicians and by then a scientist at the Institute, testified. She described Eliava as an ‘exceptionally humane and understanding man’ whose enthusiasm for scientific work was infectious. ‘Eliava was a patriot of his country, a patriot of his work …. The workers of the institute, and the whole scientific world treated his arrest as a huge loss for us, and for science.’^71^ When Antadze had compared his Institute's research programme to a lysogenic strain of bacteriophage, lying dormant until a better time, he might as well have spoken about the memory of the Institute's founder.

## Conclusion

On 19 August 1960, two dogs, Belka and Strelka, went into space aboard the Korabl’-Sputnik 2, the second Soviet orbital flight with canine passengers. Unlike their predecessor Laika, the dogs made it back to Earth safely. They were not the only passengers on the spacecraft; there were also forty mice, two rats, fruit flies, seeds, fungi, several bacterial strains, HeLa cells, and two types of bacteriophage, *Escherichia coli* T2 and *E. coli* aerogenes 1321.^72^ An extended piece in *Pravda* discussed the various aspects of the biological experiments, and described bacteriophages as ‘ultramicroscopic living creatures that parasitize bacteria and enter into complex genetic relationships with them.’^73^ The phages were there specifically for genetic experiments; and the T2 phage was chosen because it was well-characterized genetically, while there were many useful Soviet electron microscope images for the aerogenes 1321 phage revealing the process of lysis. Far from the complex and uncertain trends of the 1950s, Soviet biology entered the Space Age in alignment with international narratives in molecular biology and genetics.

This alignment represented a prioritization of a certain set of narratives on the nature of bacteriophage. As Soviet scientists participated in the global debates over the mysterious properties of phages in the 1920s and 1930s, they developed bacteriophage research as a crucial problem for materialist biology, and a promising medical intervention. During the Winter War and World War II, while most Soviet biologists favoured the enzymatic nature of phage, the language and metaphors they used in both expert and the more popular accounts endowed phage with animacy, and the tension between lively descriptions and ambiguous status maintained interest in research. Electron micrographs of phages commanded attention and fuelled debates, but were on their own insufficient to settle the issue. While multiple accounts had coexisted in the 1930s and early 1940s, the rise of Lysenkoism and political tensions of the early Cold War changed the way phages could be discussed significantly, if not as dramatically as in the case of genetics. Michurinist approaches framed phages in new ways—when treated as spores or developmental phases of bacteria, eliding the living/dead dichotomy in unexpected ways. These new narratives had political power—whereas many microbiologists remained sceptical, they had to frame their critique within the correct language if they wanted to be published.

**Figure 2.**
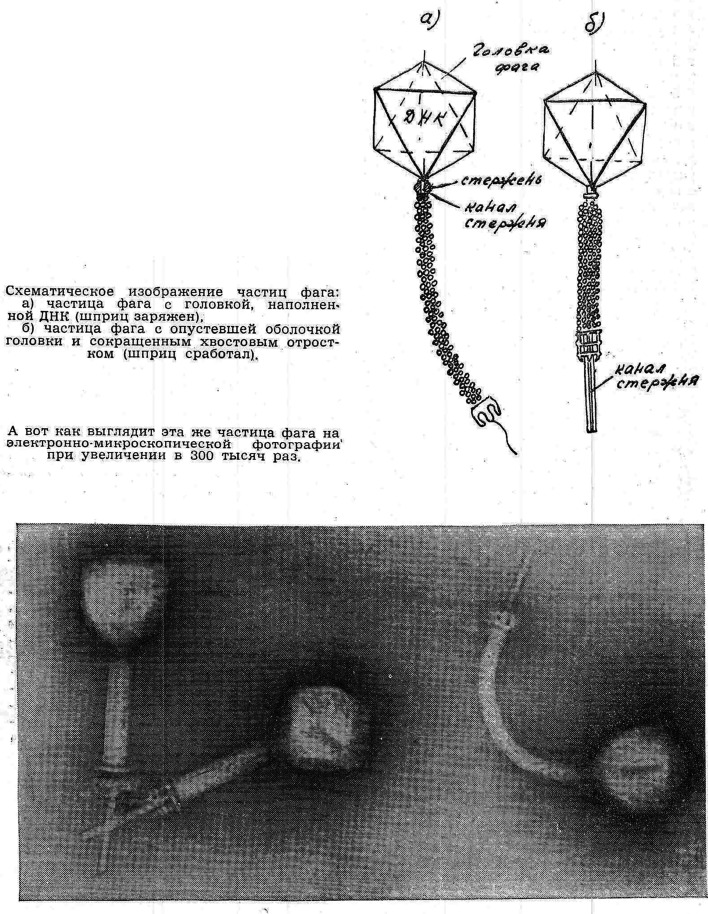
A structural diagram as a reading of an electron micrograph of bacteriophages represents a new consensus on the nature and structure of bacteriophages in post-Lysenkoist Soviet biology. From V. A. Engel'gardt, ‘Khmiia zhizni’, *Nauka i zhizn’* **29**(4), 15–18 (1962), at p. 17. The caption reads, above: ‘Schematic representation of phage particles: (a) bacteriophage particle with a head filled with DNA (the syringe is loaded); (b) phage particle with an empty head capsule and shortened tail appendage (the syringe has worked).’ Below, ‘And this is what the same phage particle looks like on an electron microscope photograph, magnified 300,000 times.’ (Reproduced courtesy of Nauka i Zhizn'.)

The more dramatic readings of bacteriophage within the framework set up by Lysenko, Lepeshinskaya, and Bosh'ian did not last long. In 1962, two years after the space flight, the flagship popular science magazine *Nauka i Zhizn’* [*Science and Life*] ran a special issue on what life meant for different branches of biology. The magazine assembled a press conference featuring major personalities in the Soviet life sciences, who made short statements.^74^ Viruses, and bacteriophages among them, attracted significant attention as a liminal case of life—whereas they reproduced, they lacked metabolism. Engels' definition of life was an important starting point, namely that *‘*life is the mode of existence of albuminous [i.e. protein] bodies, and this mode of existence essentially consists in the constant self-renewal of the chemical constituents of these bodies.’^75^ Some, like Alexander Oparin, drew on Engels to argue that metabolism was a crucial feature of life, and that viruses would not count as living since they could only be animated within a cell. Other scientists suggested that viruses were definitely living, in the same way that seeds were, and that proteins were not the exclusive molecule of life, since bacteriophages only appeared to insert nucleic acids into bacteria. Lysenko was invited to the press conference, but his comments were limited to how biological laws were irreducible to physics and chemistry—he was not given room to elaborate. Despite disagreements over the boundaries of life, a new consensus was evident—the nature of viruses and phages, their structure and mode of infection were no longer in question, and a diagram of the T4 phage, the typical ‘spaceship’ image familiar from molecular biology, cemented the accepted view (figure 2).

Discussions of life, non-life, and the boundary between them have been a continuous theme in the history of biology, but had distinct layers in the Soviet context. As Alexander Etkind has argued in his study of responses to Stalinism in Soviet and post-Soviet culture, the boundary between the living and the dead remained troubled long after the 1930s. Figures of the undead, uncanny narratives, ghosts from the past—rehabilitated but not properly mourned—haunted a whole layer of Soviet literature, cinema and visual arts.^76^ Victims of Stalinism, like Eliava and others, remained absent from official narratives until long after their formal rehabilitation, and feature as awkward absences and omissions in scientific narratives. The framework of animacy, highlighting the multiple ways of drawing boundaries between life and non-life, and the hierarchies implicit in such designations, brings forward a speculative question, hinted at in this discussion but prime for future investigation. Can we read the post-war Soviet narratives that demarcate the boundaries and meanings of biological life and highlight liminal cases such as the nature of bacteriophage, as—among other things—an attempt to process the traumatic historical experience of war and terror?

## Footnotes

1 On bacteriophages in the USSR, see Nina Chanishvili, ‘Phage therapy—history from Twort and d'Herelle through Soviet experience to current approaches’, *Adv. Virus Res*. **83**, 3–40 (2012); Dmitriy Myelnikov, ‘An alternative cure: the adoption and survival of bacteriophage therapy in the USSR, 1922–1955’, *J. Hist. Med. Allied Sci.* **73**, 385–411 (2018).

2 The phrase was attributed to Georgy Nadson; see Sofia Kazarnovskaia, *Bakteriofagiia* (Medgiz, Moscow, 1933), p. 109. I thank Charlotte Sleigh for suggesting this translation which retains the Russian rhyme (‘*sushchestvo so svoistvami veshchestva*’). All subsequent translations from Russian are my own.

3 NAG, f. 1991, *op*. 1, d. 64., l. 2–12. On phage typing, see Claas Kirchhelle, ‘The forgotten typers: the rise and fall of Weimar bacteriophage-typing (1921–1935)’, this issue.

4 Louis Campos, *Radium and the secret of life* (University of Chicago Press, Chicago, 2015).

5 Mel Y. Chen, *Animacies: biopolitics, racial matterings, and queer affect* (Duke University Press, Durham, NC, 2012).

6 On narratives in science, see Mary S. Morgan and M. Norton Wise, ‘Narrative science and narrative knowing. Introduction to special issue on narrative science’, *Stud. Hist. Phil. Sci*. *A* **62**, 1–5 (2017), and articles in that special issue. Further resources on using narrative in history and philosophy of science are provided by the Narrative Science Project at the London School of Economics, https://www.narrative-science.org (accessed 25 November 2019).

7 For an account of the earlier practices of phage research and therapy in the USSR, see Myelnikov, *op. cit.* (note 1), 392–396, 401–406.

8 William Summers, *Félix d'Herelle and the origins of molecular biology* (Yale University Press, New Haven, 1999).

9 Nikolai Gamaleia, ‘Bakterioliziny—fermenty, razrushaiushchie bakterii’, *Russ. Ark. Patol., Klin. Med. Bakteriol.* (1898); see also David Bardell, ‘An 1898 report by Gamaleya for a lytic agent specific for *Bacillus anthracis*’, *J. Hist. Med. Allied Sci*. **37**, 222–225 (1988).

10 For a summary, see Summers, *op. cit.* (note 7), 82–96.

11 Ton van Helvoort, ‘The construction of bacteriophage as bacterial virus: linking endogenous and exogenous thought styles’, *J. Hist. Biol.* **27**, 91–139 (1994).

12 Nikolai Gamaleia and O. I. Shvetsova, ‘Issledovaniia o bakteriolizinakh i bakteriofagakh’, *Gig. epidemiol.* **3**, 1–8 (1924); Nikolai Gamaleia, *Biologicheskie protsessy razrusheniia bakterii* (Gosudarstvennoe izdatel'stvo meditsinskoi literatury, Moscow, 1934), pp. 35–43.

13 Nikolai Krementsov, *Revolutionary experiments: the quest for immortality in Bolshevik science and fiction* (Oxford University Press, 2014). Krementsov borrows the ‘visionary biology’ definition from Mark Adams.

14 Raphael Falk and Antonio Lazcano, ‘The forgotten dispute: A. I. Oparin and H. J. Muller on the origin of life’, *Hist. Phil. Life Sci.* **34**, 373–390 (2012).

15 Vladimir Vernadskii, ‘Bakteriofag i skorost’ peredachi zhizni v biosfere’, *Priroda* **16**, 434–446 (1927).

16 Kazarnovskaia, *op. cit.* (note 2), p. 109.

17 Ganna Eliava-Malieva, ‘Vospominaniia’, *Rus. Klub* (Tbilisi), 2017, nos 2–6.

18 Vira Hamaliia, ‘Hnat Omelianovych Ruchko (1883–1937): Trahichna dolia vchenogo’, *Mikrobiol. Zh.* **72**, 65–74 (2010).

19 See Myelnikov, *op. cit.* (note 1).

20 Scott Montgomery, ‘Codes and combat in biomedical discourse’, *Scient. Cult.* **2**, 341–390 (1991); Alfred Tauber, *The immune self: theory or metaphor?* (Cambridge University Press, 1994); Michael Worboys, *Spreading germs: disease theories and medical practice in Britain, 1865–1900*, pp. 6–7, 281 (Cambridge University Press, 2000); Jon Turney, ‘Beyond cell wars’, *Aeon*, 28 March 2016, available at https://aeon.co/essays/why-we-should-guard-against-military-notions-of-immunity (accessed 9 August 2019).

21 A. P. Tsulukidze, *Kratkoe nastavlenie po primeneniiu bakteriofaga pri lechenii ran* (Gruzmedgiz, Tbilisi, 1942), pp. 7–9.

22 M. P. Pokrovskaia, L. S. Kaganova, M. A. Morozenko, A. G. Bulgakova, E. E. Skatsenko, *Lechenie ran bakteriofagom*, pp. 3–4 (Medgiz, Moscow, 1941).

23 *Ibid*., p. 12.

24 *Ibid*., pp. 12–13.

25 *Ibid.*, p. 9.

26 E. Kramskoi [Sergei Beliaev], *Kovarnoe oruzhie* (Detgiz, Moscow, 1942). Sergei Beliaev is unrelated to the more celebrated Soviet science fiction novelist Alexander Beliaev.

27 The tropes of heroic bacteriology were common for literary accounts of the 1920s, most notably Sinclair Lewis's *Arrowsmith* (Harcourt Brace, New York, 1925), dealing with a thinly veiled discovery of bacteriophage, with the protagonist, Martin Arrowsmith, based partly on d'Herelle. See William C. Summers, ‘On the origins of the science in *Arrowsmith*: Paul de Kruif, Felix d'Herelle, and phage’, *J. Hist. Med. Allied Sci.* **46**, 315–332 (1991). *Arrowsmith* was translated into Russian in the year of its publication by Mikhail P. Chekhov, the younger brother and biographer of Anton Chekhov: Sinkler L'iuis, *Martin Arrousmit*, trans. M. P. Chekhov (Mysl’, Leningrad, 1925). Two further translations appeared in 1929 and 1936; S. L'iuis, *Errousmit*, trans. L. L. Domer, in S. L'iuis, *Sobranie sochinenii*, v. 2 (Gosudarstvennoe izdatel'stvo, Moscow, 1929); S. L'iuis, *Erousmit*, trans. Nadezhda Vol'pin (Goslitizdat, Moscow, 1936).

28 Beliaev's following work, a novel titled *The adventures of Samuel Pingle* [*Prikliucheniia Semiuelia Pinglia*] (Detgiz, Moscow, 1945)—also highlights his interest in microbiology. Its protagonist has learned to select filterable viruses in ways that can modify an organism's protein composition, allowing people to change appearance.

29 Summers, *op. cit.* (note 7), pp. 191–192.

30 Moisei Fisher, *Bakteriofag—pozhiratel’ mikrobov*, p. 5 (Leningradskii dom sanitarnogo prosvescheniia, Leningrad, 1945).

31 *Ibid*., pp. 6, 8, 10.

32 van Helvoort, *op. cit.* (note 10); Angela Creager, *The life of a virus: tobacco mosaic virus as an experimental model, 1930–1965*, pp. 185–248 (University of Chicago Press, Chicago, 2001); Neeraja Sankaran, ‘The bacteriophage, its role in immunology: how Macfarlane Burnet's phage research shaped his scientific style’, *Stud. Hist. Phil. Biol. Biomed. Sci.* **41** (2010); Lily Kay, *The molecular vision of life: Caltech, the Rockefeller Foundation, and the rise of the new biology*, pp. 243–256 (Oxford University Press, 1993)*.*

33 Ton van Helvoort and Neeraja Sankaran, ‘How seeing became knowing: the role of the electron microscope in shaping the modern definition of viruses’, *J. Hist. Biol.* (2018), https://doi.org/10.1007/s10739-018-9530-2 (accessed 31 January 2019).

34 *Ibid.*; Nicholas Rasmussen, *Picture control: the electron microscope and the transformation of biology in America, 1940–1960* (Stanford University Press, 1999).

35 The literature on history and sociology of images and visual arguments in science is vast, but for bacteriophages see Alberto Cambrosio, Daniel Jacobi, and Peter Keating, ‘Phages, antibodies and “demonstration”’, *Hist. Phil. Life Sci.* **30**, 131–157; van Helvoort and Sankaran, *op. cit.* (note 32).

36 Boris Klein, ‘Mikroby v elektronnom “sverkhmikroskope”’, *Nauk. Zhiz.,* no. 11–12, 35–38 (1945).

37 Ibid.

38 Anatoliy Kriss, ‘Sushchnost’ bakteriofagii’, *Priroda* **34**(1), 36–48 (1945).

39 Myelnikov, *op. cit.* (note 1).

40 A. Savin, ‘Vglub’ mikromira’, *Tekhnika—molodëzhi*, September 1949, pp. 11–14 (1949), p. 13.

41 Chen, *op. cit.* (note 4), p. 2.

42 Nikolai Krementsov, ‘“In the shadow of the bomb:” US–Soviet biomedical relations in the early Cold War, 1944–1948’, *J. Cold War Stud.* **9**, 41–67 (2007); *The cure: a story of cancer and politics from the annals of the Cold War* (University of Chicago Press, Chicago, 2002)*.* On the Cold War and Soviet biomedicine, see also Anna Geltzer, ‘In a distorted mirror: the Cold War and U.S.–Soviet biomedical cooperation and (mis)understanding, 1956–1977’, *J. Cold War Stud.* **14**, 39–63 (2012); William DeJong-Lambert and Nikolai Krementsov, ‘On labels and issues: the Lysenko controversy and the Cold War’, *J. Hist. Biol.* **45**, 373–388 (2012) and articles in this special issue, *The Lysenko controversy and the Cold War.* On the US side of Cold War scientific diplomacy, see, for example, John Krige, *American hegemony and the postwar reconstruction of science in Europe* (MIT Press, Cambridge, MA, 2006); Audra J. Wolf, *Freedom's laboratory: the Cold War struggle for the soul of science* (Johns Hopkins University Press, Baltimore, 2018).

43 Myelnikov, *op. cit.* (note 1).

44 Much has been written on Lysenko and Lysenkoism; for a historiographic overview, see Nikolai Krementsov and William deJong-Lambert, ‘“Lysenkoism” redux: introduction’, in *The Lysenko controversy as a global phenomenon*, vol. 1 (ed. Nikolai Krementsov and William deJong-Lambert), pp. 1–34 (Palgrave, Cham, 2017). On the longer-term impact of Lysenkoism and Lamarkian thinking on Russian biology, see Loren Graham, *Lysenko's ghost: epigenetics and Russia* (Harvard University Press, Cambridge, MA, 2016). On the decline of Lysenkoism, see Michael D. Gordin, ‘Lysenko unemployed: Soviet genetics after the aftermath’, *Isis* **109**, 56–78 (2018).

45 ‘O polozhenii v biologicheskoi nauke’, Stenographic report of the VASKhNIL session of 31 July–7 August 1948, available online at http://lib.ru/DIALEKTIKA/washniil.txt (accessed 11 February 2019). After Lysenko's victory, Rapoport was expelled from the Communist party and worked in geological expeditions until 1957.

46 *Ibid*. Muromtsev, a veterinary microbiologist, had run a *sharashka*, or prison research laboratory within the Gulag system, during the terror and the war; Valery Soifer, *Stalin i moshenniki v nauke*, pp. 361–362 (Dobrosvet, Moscow, 2012).

47 On the place of Michurin's figure in Lysenkoism, see Douglas R. Weiner, ‘The roots of “Michurinism:” transformist biology and acclimatization as currents in the Russian life sciences’, *Ann. Sci.* **42**, 243–60 (1985); Nikolai Krementsov, *Stalinist science*, pp. 158–225 (Princeton University Press, 1997); Krementsov and deJong-Lambert, *op. cit.* (note 41); Pat Simpson, ‘Lysenko's “Michurinism” and art at the Moscow Darwin Museum 1935–1964, in *The Lysenko controversy as a global phenomenon*, vol. 1 (ed. Nikolai Krementsov and William deJong-Lambert) (Palgrave, Cham, 2017), pp. 129–75.

48 Sergei Muromtsev, *Problemy sovremennoi mikrobiologii v svete michurinskogo ucheniia* (Pravda, Moscow, 1950)*.*

49 On Lepeshinskaya, see Valerii Soifer, *Vlast’ i nauka: istoriia razgroma genetiki v SSSR*, pp. 463–477 (Lazur’, Moscow, 1993).

50 *Ibid*., pp. 500–509.

51 Gevork Bosh'ian, *O prirode virusov i mikrobov*, 2nd edn, pp. 122–123 (Medgiz, Moscow, 1950).

52 V. A. Krestovnikova, V. I. Zhurbina and N. B. Izmailova, ‘K vorposu o prirode bakteriofaga’, *Mikrobiologiia* **21**, 721–734 (1952).

53 Soifer, *op. cit.* (note 48), p. 505, explains this distancing on Lysenko's part by Bosh'ian never citing him or playing proper homage to Michurinist biology.

54 Aleksandr Kriviskii, ‘Biologicheskaia priroda bakteriofaga’, *Priroda* no. 10, 45–57 (1952),

55 ‘Agrobiologiia Michurina–Lysenko primenitel'no k mikrobiologii’, papers of the Tbilisi Institute of Vaccines and Sera, National Archives of Georgia (NAG), f. 1991, op. 1, d. 29.

56 *Ibid.*, f. 1991, *op*. 1, d. 35, l. 49.

57 See Myelnikov, *op. cit*. (note 1).

58 NAG, f. 1991, *op*. 1, d. 64, l. 119.

59 *Ibid*. Some journals did become available in the 1950s. Polish and Hungarian scientific journals appeared in 1952, and *Journal of Bacteriology* began to be available in 1956. Moreover, the *Reference Journal* (*Referativnyi Zhurnal*), which included Russian translations of the abstracts published in major foreign scientific journals, was supplied to the library permanently in the mid-1950s.

60 Krestovnikova *et al.*, *op. cit.* (note 51).

61 NAG, f. 1991, *op*. 1, d. 35, l. 50.

62 Yurii Zeifman, interviewed by Nikolai Gladkikh, ‘Penicillinovoe delo’, *Mezhdunarodnoe Obshchestvo “Memorial”*, nos 93–94, 30 October 2010; available online at http://old.memo.ru/2010/02/04/penicillin.htm (accessed 9 August 2019). See also Mikhali Shifrin, *100 rasskazov iz istorii meditsiny* (Alpina Digital, Moscow, 2019), pp. 352–353.

63 ‘Penitsillin—russkoe otkrytie’, *Pravda*, 17 March 1948, p. 3.

64 Vladimir Antadze, ‘Osnovnye zadachi izucheniia problemy bakteriofagii i puti ikh resheniia’, in *Bakteriofagiia: sbornik trudov mezhinstitutskoi nauchnoi konferentsii, sostoiavsheisia v Tbilisi 26–29 oktiabria 1955 goda*, pp. 5–17 (Gruzmedgiz, Tbilisi, 1957).

65 T. A. Kursanova, ‘Sud'ba uchenogo v kontekste ideologicheskoi bor'by v Akademii Nauk SSSR. K 150-letiiu akademika G. A. Nadsona (1867–1939)’, *Istoriko-biologicheskie issledovaniia* **9**, 54–79 (2017).

66 Iraklii Khvadagiani, ‘Zhertvy politicheskikh repressii v Gruzii: otnoshenie obshchestva i protsess reabilitatsii’, in *Reabilitatsiiia i pamiat’: otnoshenie k zhertvam sovetskikh politicheskikh repressii v stranakh byvshego SSSR* (ed. L. S. Eremina), pp. 152–192 (Memorial, Moscow, 2016); available at www.memo.ru/media/uploads/2017/03/02/reabilitacia.pdf (accessed 26 May 2019).

67 Oleg Khlevniuk, ‘Kremlin—Tbilisi: purges, control and Georgian nationalism in the first half of the 1950s’, in *Georgia after Stalin: nationalism and Soviet power* (ed. Timothy K. Blauvelt and Jeremy Smith), pp. 13–31 (Routledge, London, 2015).

68 D. P. Shrayer, ‘Felix d'Herelle in Russia’, *Bull. Inst. Pasteur* **94**, 91–96 (1996).

69 A. S. Kriviskii, *Virusy protiv mikrobov*, p. 34 (Medgiz, Moscow, 1962).

70 Eliava's name appeared on a local album published by the Tbilsi Institute in 1967, which credited Eliava as the first director who ‘played a major role in the institute's creation’ and a ‘pioneer and foundational figure for the problem of bacteriophage and introducing bacteriophage into healthcare practice in the Soviet Union’—yet it is unclear whether this publication ever circulated beyond the Institute. An album titled *Tbilisskii Nauchno-Issledovatel'skii Institut Vaktsin i Syvorotok,* printed in 1967, alongside Georgadze's archival research notes was kindly shared with me by Nina Chanishvili of the Eliava Institute.

71 Archives of the Ministry of the Internal Affairs of Georgia (Tbilisi), Budu Mdivani case, *tom* 5, pp. 108–109.

72 ‘Programma nauchnykh issledovanii uspeshno vypolnena: podrobnosti o “passazhirakh” korablia’, *Pravda,* 23 August 1960, p. 1. Contemporary designations identify both bacterial phage targets as *Bact. coli*. On Soviet space dogs, see Amy Nelson, ‘Cold War celebrity and the courageous canine scout: the life and times of the Soviet space dogs’, in *Into the cosmos: a cultural history of the Soviet Space Age* (ed. James T. Andrews and Asif Siddiqi), pp. 133–155 (University of Pittsburgh Press, 2011).

73 ‘Programma nauchnykh issledovanii’, *op. cit.* (note 72), p. 1.

74 ‘Nauka o zhizni i zhisn’’, *Nauk. Zhiz* **29**(4), 1–73 (1962).

75 Frederick Engles, *Anti-Dühring*, in Karl Marx and Frederick Engels, *Marx & Engels Collected Works. Vol. 25: Engels: dialectics of nature* (Lawrence & Wishart, London, 1987), p. 76.

76 Alexander Etkind, *Warped mourning: stories of the undead in the land of the unburied* (Stanford University Press, 2013). See also Sergei Prozorov, *Biopolitics of Stalinism: ideology and life in Soviet socialism* (Edinburgh University Press, 2016).

